# Effect of memantine, an anti-Alzheimer’s drug, on rodent microglial cells in vitro

**DOI:** 10.1038/s41598-021-85625-4

**Published:** 2021-03-17

**Authors:** Toru Murakawa-Hirachi, Yoshito Mizoguchi, Masahiro Ohgidani, Yoshinori Haraguchi, Akira Monji

**Affiliations:** 1 Department of Psychiatry, Faculty of Medicine, Saga University, 5-1-1 Nabeshima, Saga, 849-8501 Japan; 2 Department of Integrative Anatomy, Nagoya City University Graduate School of Medical Sciences, Nagoya, 467-8601 Japan; 3 Department of Neuropsychiatry, Graduate School of Medical Sciences, Kyushu University, 3-1-1 Maidashi, Higashi-ku, Fukuoka, 812-8582 Japan

**Keywords:** Cell biology, Immunology

## Abstract

The pathophysiology of Alzheimer’s disease (AD) is related to neuroinflammatory responses mediated by microglia. Memantine, an antagonist of N-methyl-d-aspartate (NMDA) receptors used as an anti-Alzheimer’s drug, protects from neuronal death accompanied by suppression of proliferation and activation of microglial cells in animal models of AD. However, it remains to be tested whether memantine can directly affect microglial cell function. In this study, we examined whether pretreatment with memantine affects intracellular NO and Ca^2+^ mobilization using DAF-2 and Fura-2 imaging, respectively, and tested the effects of memantine on phagocytic activity by human β-Amyloid (1–42) phagocytosis assay in rodent microglial cells. Pretreatment with memantine did not affect production of NO or intracellular Ca^2+^ elevation induced by TNF in rodent microglial cells. Pretreatment with memantine also did not affect the mRNA expression of pro-inflammatory (TNF, IL-1β, IL-6 and CD45) or anti-inflammatory (IL-10, TGF-β and arginase) phenotypes in rodent microglial cells. In addition, pretreatment with memantine did not affect the amount of human β-Amyloid (1–42) phagocytosed by rodent microglial cells. Moreover, we observed that pretreatment with memantine did not affect 11 major proteins, which mainly function in the phagocytosis and degradation of β-Amyloid (1–42), including TREM2, DAP12 and neprilysin in rodent microglial cells. To the best of our knowledge, this is the first report to suggest that memantine does not directly modulate intracellular NO and Ca^2+^ mobilization or phagocytic activity in rodent microglial cells. Considering the neuroinflammation hypothesis of AD, the results might be important to understand the effect of memantine in the brain.

## Introduction

Approximately 46.8 million people suffer from dementia, with an accompanying medical cost estimated at 818 billion dollars. Furthermore, the accumulation of β-Amyloid (1–42) is supposed to begin from 10 to 15 years before the onset of cognitive symptoms in patients suffering from Alzheimer’s disease (AD)^1^. In AD, β-Amyloid (1–42) directly stimulates microglia to release both pro-inflammatory cytokines (such as TNFα) and nitric oxide (NO)^2^ and promotes neuroinflammation resulting in neurodegeneration^3^. In addition, microglial senescence, typified by a decrease in phagocytic ability, is recently supposed to underlie mechanisms of both aging and AD^4^. Recently, many outstanding reports focus on triggering receptor expressed on myeloid cell 2 (TREM2) and/or DNAX-activating protein of 12 kDa (DAP12), which are important for maintaining the phagocytic ability of microglia^5,6^.

For the treatment of patients suffering from AD, we expect the appearance of disease-modifying drugs, which are fundamental treatments that suppress the progression of AD pathology^7,8^. However, we need to continue to use currently available symptomatic drugs (i.e. donepezil, galantamine, rivastigmine and memantine) for the treatment of patients suffering from AD, especially when AD is accompanied by behavioral and psychological symptoms of dementia (BPSD)^9,10^.

Memantine, an antagonist of N-methyl-d-aspartate receptors (NMDARs), has beneficial roles in the treatment of patients with moderate to severe AD because of its blockade of extra-synaptic NMDARs, which are activated by excess glutamate^11^. Microglia also express NMDARs, and application of high concentrations of NMDA release pro-inflammatory cytokines to induce death in cortical neurons^12^. In animal models of AD, memantine has been reported to protect from neuronal death accompanied by suppression of proliferation and the activation of microglial cells^13,14^, As a direct effect of memantine on microglial cells, Tsai et al. reported that memantine suppresses the amplitude of inwardly rectifying K^+^ currents, resulting in the depolarization of rodent microglial cells^15^. However, it remains to be tested whether pretreatment with memantine directly affects the intracellular NO and Ca^2+^ mobilization and/or phagocytic activity in rodent microglial cells. The protocol of experiments in this study was referred to in our previous report suggesting that donepezil, another anti-Alzheimer’s drug, has a direct effect on rodent microglial function^16^.

## Results

### Pretreatment with memantine did not affect production of NO or intracellular Ca^2+^ elevation induced by TNF in rodent microglial cells (rat HAPI, mouse 6–3 and mouse primary microglial cells)

**Figure 1:**
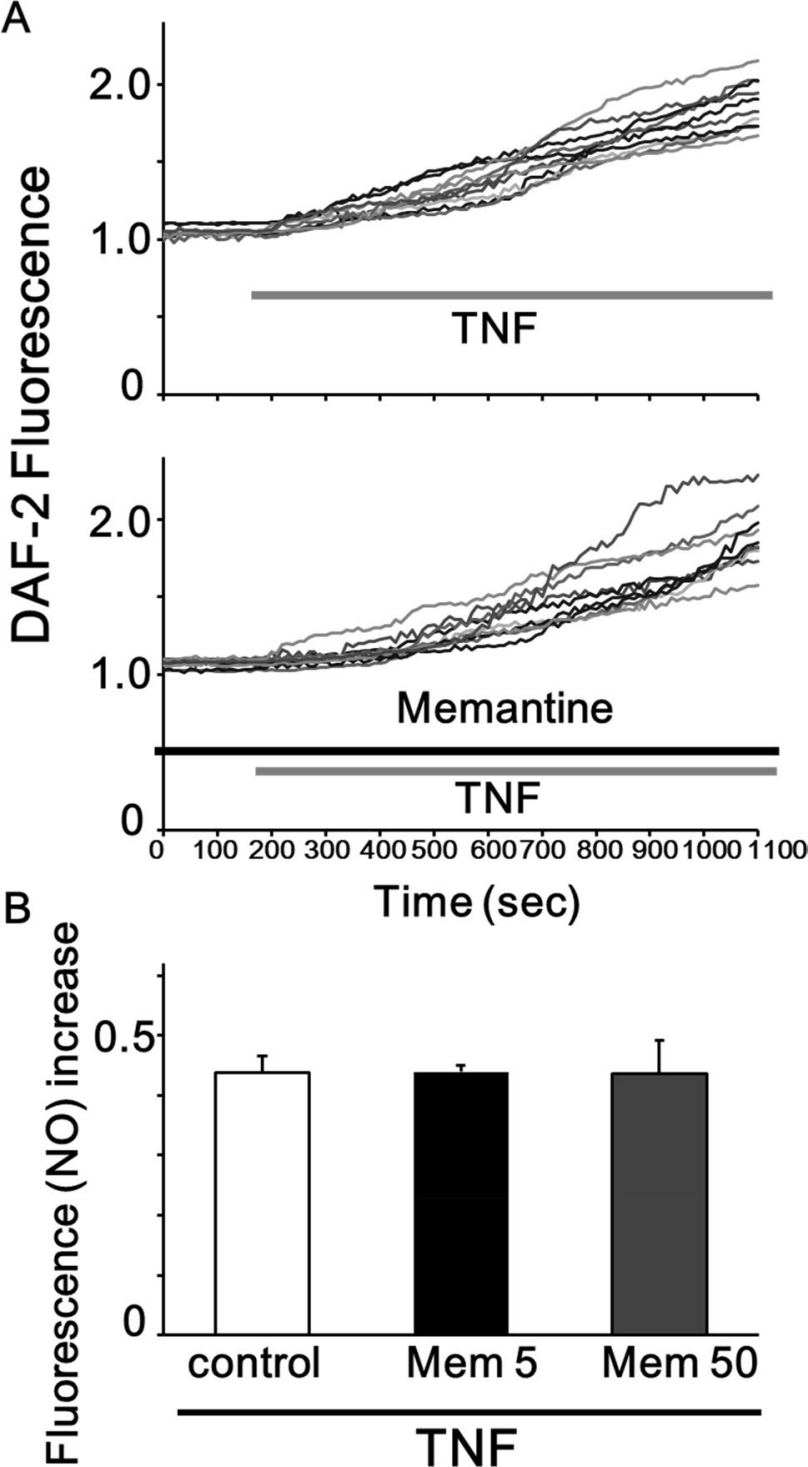
Pretreatment with memantine did not affect the production of NO induced by TNF in rat HAPI microglial cells. (**A**) Ten representative traces showing the treatment of 0.1 ng/mL TNF induced the increase in the DAF-2 fluorescence (upper) and 12 h pretreatment with 5 μM memantine did not affect the TNF-induced increase in the DAF-2 fluorescence in rat HAPI microglial cells (lower). (**B**) Bar graphs showing that pretreatment with memantine did not the production of NO induced by TNF treatment. Pretreatment of memantine did not affect the amount of TNF-induced increase in the DAF-2 fluorescence at 15 min after a TNF-treatment in rat HAPI microglial cells (0.437 ± 0.024, n = 135 cells in control; 0.439 ± 0.023, n = 145 cells in 5 µM memantine; p = 0.48).

We examined whether TNF induces intracellular NO mobilization in rat HAPI microglial cells using DAF-2 imaging. We observed that an application of TNF (0.1 ng/mL) induced a gradual increase in DAF-2 fluorescence which reflects endogenously produced NO in rat HAPI microglial cells (n = 135 cells; Fig. 1a upper)^16,17^. In addition, in the presence of L-N6-(1-iminoethyl)lysine (L-NIL; 50 µM), a membrane-permeant selective inhibitor of inducible nitric oxide synthase (iNOS)^20^, TNF did not elevate the DAF-2 fluorescence in rat HAPI microglial cells (n = 60 cells; data not shown). We also observed that the increase in intracellular DAF-2 fluorescence was sustained for more than 50 min even after the TNF washout. Because the reaction between NO and DAF-2 is shown to be irreversible, the DAF-2 fluorescence level has been reported to reflect the total amount of NO produced in the cells^18,19^.

We next measured the effect of pretreatment with memantine (5 µM; 12 h) on the TNF-induced production of intracellular NO in rat HAPI microglial cells. In rat HAPI microglial cells, which were pretreated with memantine, TNF (0.1 ng/mL) induced a gradual increase in the DAF-2 fluorescence (Fig. 1a lower). On the other hand, we observed pretreatment of memantine did not affect the amount of TNF-induced increase in the DAF-2 fluorescence at 15 min after a TNF-treatment in rat HAPI microglial cells (0.437 ± 0.024, n = 135 cells in control; 0.439 ± 0.023, n = 145 cells in 5 µM memantine; p = 0.48; Fig. 1b). We also observed that pretreatment with 50 µM memantine did not affect the TNF-induced production of NO (0.436 ± 0.046, n = 47 in memantine; p = 0.49; Fig. 1b). These suggest that memantine pretreatment did not affect the TNF-induced production of NO.

**Figure 2:**
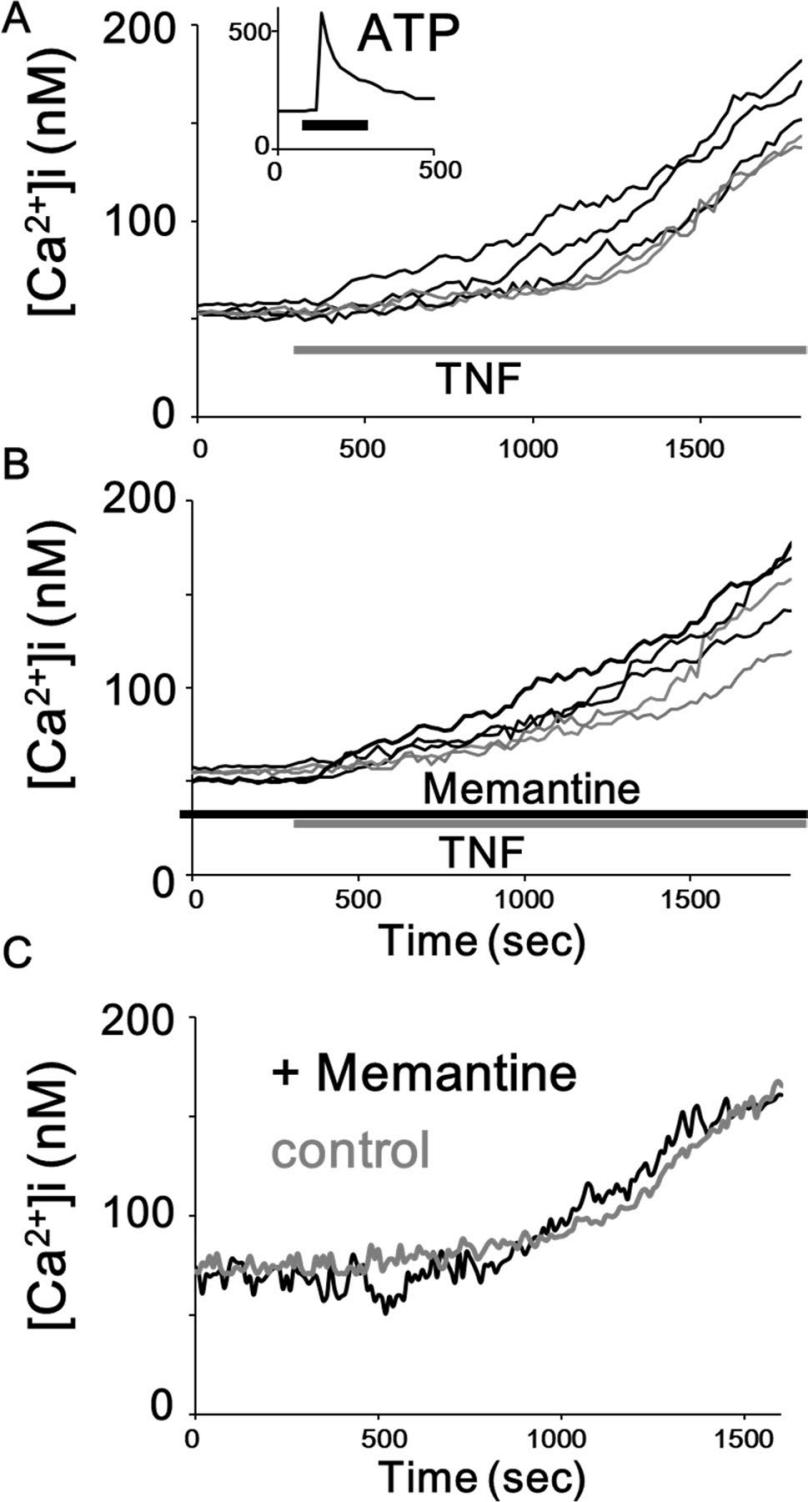
Pretreatment with memantine did not affect the elevation of [Ca^2+^]i induced by TNF in rodent microglial cells. (**A**) Five representative traces showing a treatment of 3 ng/mL TNF-induced sustained increase in [Ca^2+^]i in mouse primary microglial cells. (**A**, inset) The inset shows a 100 μM ATP-induced transient increase in [Ca^2+^]i in mouse primary microglial cells. The average trace of 10 [Ca^2+^]i traces in response to ATP is shown. (**B**) Five representative traces showing pretreatment with memantine did not affect the elevation of [Ca^2+^]i induced by TNF in mouse primary microglial cells. (**C**) Average traces of 5 [Ca^2+^]i traces showing a treatment of 3 ng/mL TNF-induced sustained increase in [Ca^2+^]i in mouse 6–3 microglial cells. Pretreatment with memantine did not affect the elevation of [Ca^2+^]i induced by TNF in mouse 6–3 microglial cells.

Next, we observed that TNF (3 ng/mL) induces a sustained elevation of [Ca^2+^]i in mouse primary microglial cells (Fig. 2a) as previously^16,17^. On contrary, an application of ATP (100 µM) rapidly elevates [Ca^2+^]i in mouse primary microglial cells (Fig. 2a, inset). We next examined whether pretreatment with memantine has any effects on the sustained elevation of [Ca^2+^]i. induced by TNF. We pretreated mouse primary microglial cells with memantine (5 µM; 12 h). We observed that memantine could not affect the elevation of [Ca^2+^]i induced by TNF in mouse primary microglial cells (92.8 ± 7.7 nM, n = 137 cells in control; 88.3 ± 8.9 nM, n = 136 cells in memantine; p = 0.35; Fig. 2b). We also observed that pretreatment with memantine did not affect the TNF-induced elevation of [Ca^2+^]i in mouse 6–3 microglial cells (22.1 ± 18.9 nM, n = 34 cells in control; 20.4 ± 14.2 nM, n = 27 cells in memantine; p = 0.47; Fig. 2c). These results suggest that memantine did not have effects on the TNF-induced elevation of [Ca^2+^]i in rodent microglial cells.

### Pretreatment with memantine did not affect the mRNA expression in pro- or anti-inflammatory phenotypes in rodent microglial cells (mouse primary microglial cells)

**Figure 3:**
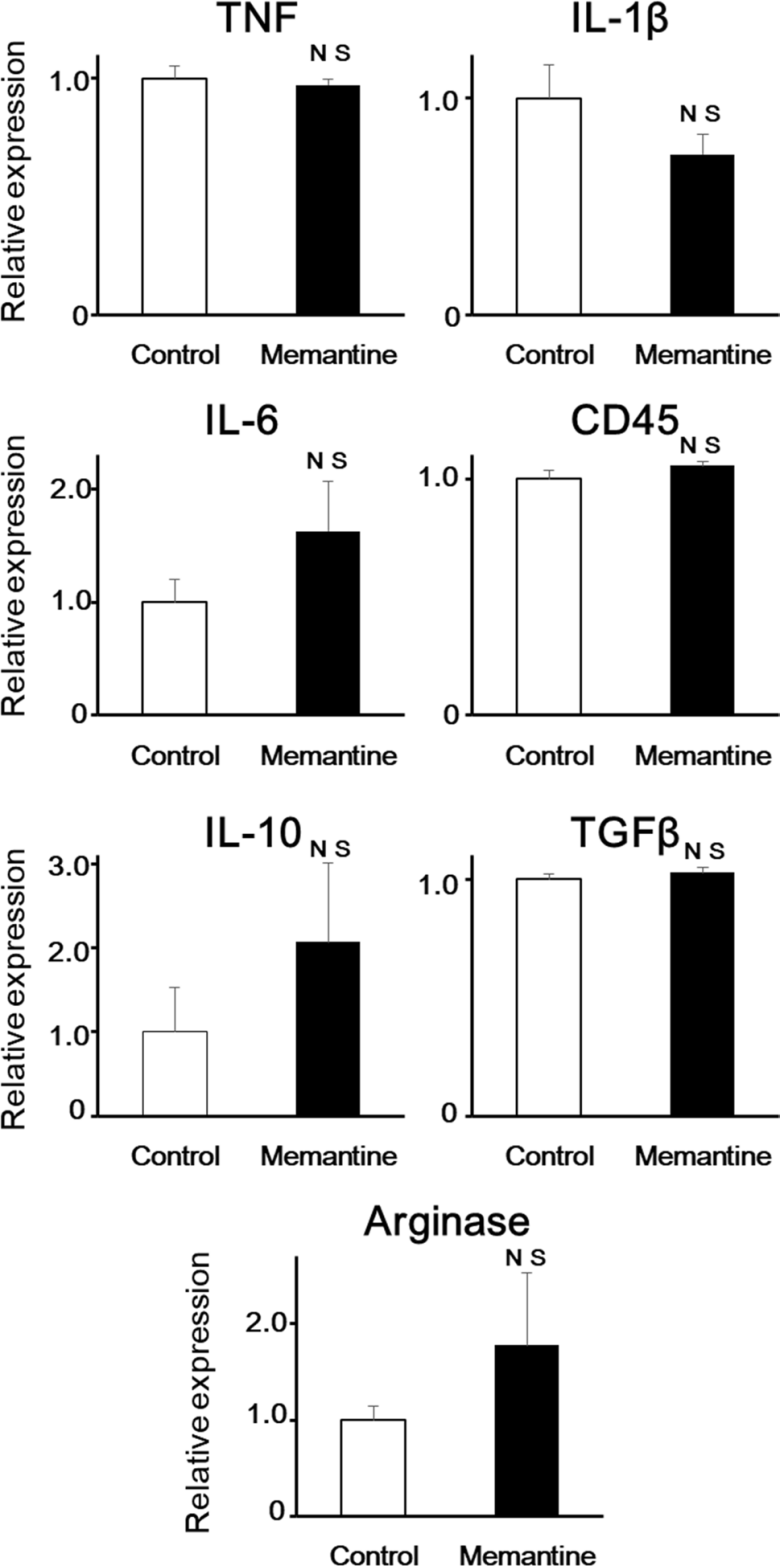
Pretreatment with memantine did not affect the mRNA expression of both pro- and anti-inflammatory phenotypes in mouse primary microglial cells. Bar graphs showing the effects of pretreatment with memantine on mRNA expression of TNF, IL-1β, IL-6, CD45, IL-10, TGF-β, and arginase in mouse primary microglial cells. *NS* not significant vs control.

In mouse primary microglial cells, we observed that memantine (5 µM; 12 h) did not have significant effects on the expressed level of mRNA of TNF, IL-1β, IL-6 and CD45 which represent pro-inflammatory markers using qRT-PCR. Memantine did not have significant effects on the expressed level of mRNA of IL-10, TGF-β and arginase which represent anti-inflammatory markers (Fig. 3).

### Pretreatment with memantine did not affect phagocytic activity of rodent microglial cells (mouse primary microglial cells)

**Figure 4:**
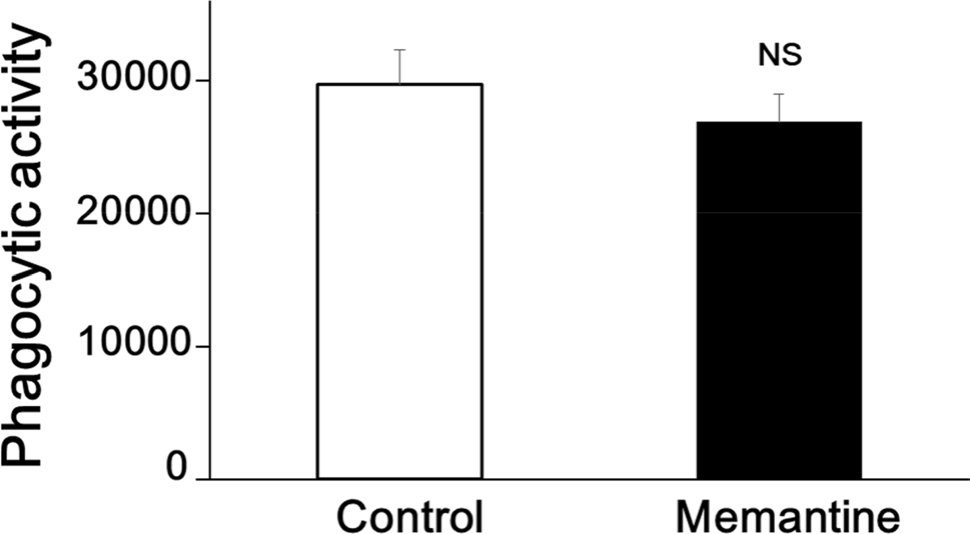
Pretreatment with memantine did not affect phagocytic activity of mouse primary microglial cells. Bar graph showing pretreatment of 5 μM memantine for 12 h did not affect the amount of β-Amyloid (1–42) phagocytosed by mouse primary microglial cells (n = 65 cells in control; n = 61 cells in memantine from 4 independent experiments each).

We next investigated whether pretreatment of memantine affects the phagocytic activity of mouse primary microglial cells. We observed that pretreatment of 5 µM memantine for 12 h did not affect the amount of β-Amyloid (1–42) phagocytosed by mouse primary microglial cells (n = 65 cells in control; n = 61 cells in memantine from 4 independent experiments each; Fig. 4). These suggest that pretreatment with memantine did not affect the phagocytic activity of rodent microglial cells.

### Effects of pretreatment with memantine on the expression of phagocytosis-related proteins in rodent microglial cells (mouse 6–3 microglial cells)

**Figure 5:**
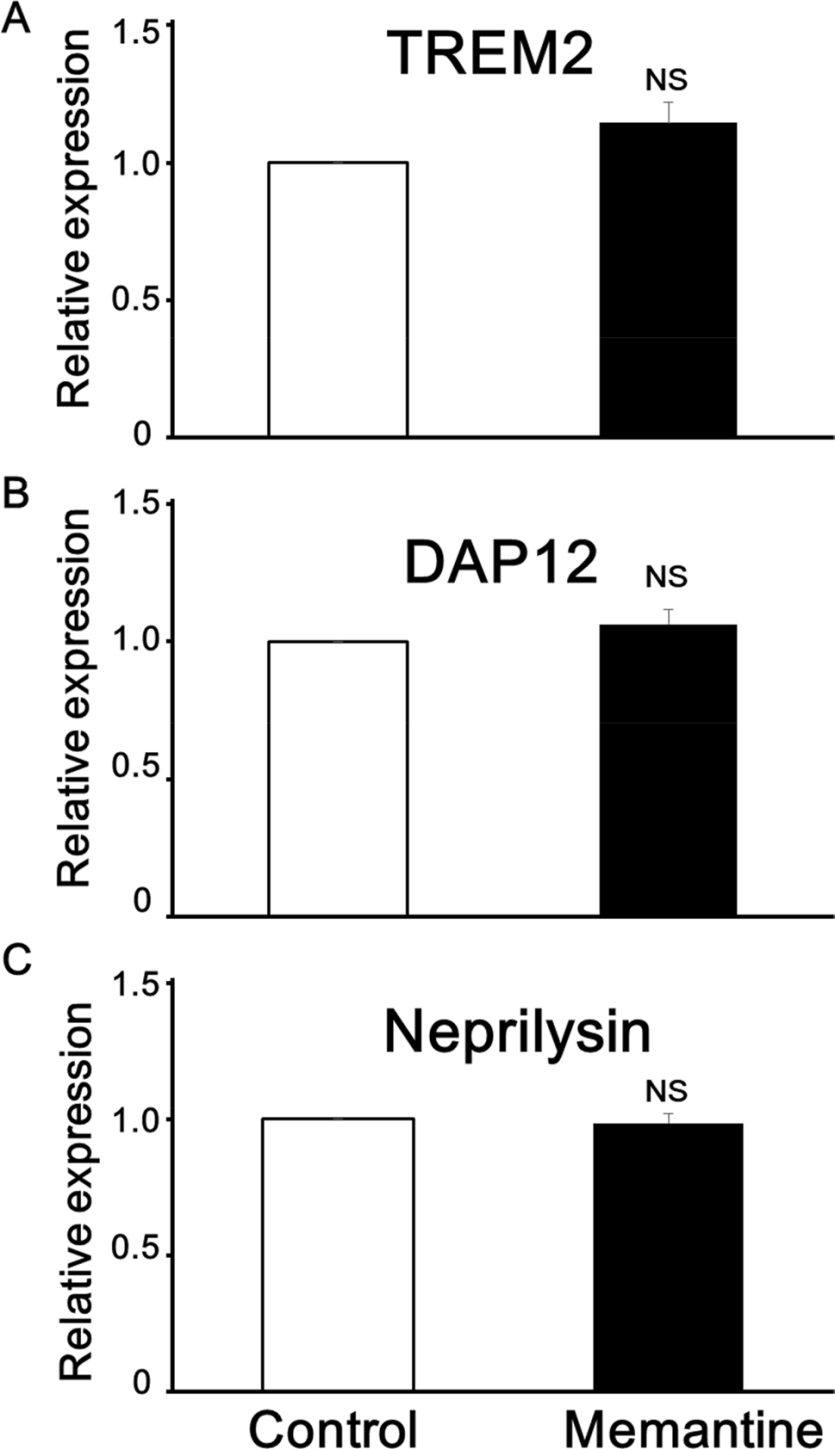
Effects of pretreatment with memantine on the expression of phagocytosis-related proteins in mouse 6–3 microglial cells. Bar graphs showing that pretreatment of 5 μM memantine for 12 h did not affect the amount of TREM2 (**A**), DAP12 (**B**) and neprilysin (**C**) expressed on mouse 6–3 microglial cells (n = 9 for TREM2, DAP12 and neprilysin from 9 independent experiments each).

We next examined whether pretreatment of memantine affects the related proteins, which mainly function in the phagocytosis of mouse 6–3 microglial cells using flow cytometry. We observed that pretreatment of 5 µM memantine for 12 h did not affect the amount of both TREM2 and DAP12, both of which are important for the phagocytic activity of microglia, expressed on mouse 6–3 microglial cells (n = 9 for TREM2 and n = 9 for DAP12 from 9 independent experiments each; Fig. 5a,b). In addition, we observed that pretreatment of 5 µM memantine for 12 h did not affect the amount of neprilysin, which is a β-Amyloid (1–42)-degrading enzyme important for the clearance of β-Amyloid (1–42) by microglia, expressed on mouse 6–3 microglial cells (n = 9 from 9 independent experiments; Fig. 5c). Moreover, we observed that pretreatment with memantine did not affect other eight proteins which mainly function in the phagocytosis, degradation of β-Amyloid (1–42) and/or intercellular signaling in mouse 6–3 microglial cells (Table 1). Specifically, pretreatment with memantine did not affect the amount of any of the following proteins including CX3CR1, CR3(CD11b/c), CD68, Dectin-1/Clec7a, Prostaglandin E synthase (PTGEs), Suppressor of cytokine signaling 3 (Socs3), ADAM10 and ADAM17. However, we observed that pretreatment with memantine significantly increased the amount of expression of A disintegrin and metalloproteinase with thrombospondin motifs 4 (ADAMTS4) and ATP binding cassette subfamily a member 7 (ABCA7) in mouse 6–3 microglial cells (Table 1).

**Table 1:** Effects of pretreatment with memantine on the expression of phagocytosis-related proteins in mouse 6–3 microglial cells measured using flow cytometry.

Effects of memantine	Function in microglial cells	References
**Potentiation**		
ABCA7	ABCA7 is important for microglial phagocytic activity. Deletion of ABCA7 in AD mouse exacerbates cerebral Aβ plaque load	^28,29^
ADAMTS4	ADAMTS4 increases the number of microglia expressing arginase-1, a marker of anti-inflammatory functions in the ischemic brain	^27^
**No effect**		
TREM2	TREM2 promotes proliferation and enhances phagocytosis of microglia, while recent reports suggesting a complex action of TREM2 on inflammatory processes because of both pro- or anti-inflammatory properties	^6,23^
DAP12	Adaptor protein gets phosphorylated, recruits SYK, and activates intracellular pathways such as PI3K and/or MAPK when binding to TREM2 ligands	^24,25^
Neprilysin	Microglia can phagocytose Aβ and also clear Aβ by degradation via Aβ degrading proteases, neprilysin	^26^
CX3CR1	Microglial activity is restrained by fractalkine (CX3CL1) derived from neurons and cognate receptors on microglia, CX3CR1, in normal brain	^42^
CR3(CD11b/c)	CR3 is surface complement receptor involved in clearance of weak synapses mediated by microglial phagocytosis	^43^
CD68	CD68, a lysosomal/endosomal associated membrane glycoprotein, is a marker for either activated and/or senescent microglia	^44^
Dectin-1/Clec7a	Dectin-1/Clec7a is a pattern recognition receptor involved in the microglial phagocytosis of beta-glucan particles	^45^
PTGEs (Prostaglandin E synthase)	PTGEs produces eicosanoid prostaglandin E2 (PGE2) which plays important roles in neuroinflammation and AD brain	^46^
Socs3 (suppressor of cytokine signaling 3)	SOCS3 suppresses JAK/STAT3 pathway resulting in cytokine signaling and has anti-inflammatory roles in the brain of AD mouse	^47,48^
ADAM10	ADAM10, one of four loci recently identified as risk genes of AD, is expressed in microglia and it cleaves CX3CL1 into a secreted form to remodel synapses	^49,50^
ADAM17	ADAM17 inhibits clearance of apoptotic cells by microglia while it potentiates cleavage of amyloid precursor protein resulting in reduction of Aβ generation	^51,52^
**Suppression**		
None		

### Effects of pretreatment with MK801, another antagonist of NMDARs, on production of NO, intracellular Ca^2+^ elevation and phagocytic activity in rodent microglial cells (mouse 6–3 and mouse primary microglial cells)

Mouse primary microglial cells are reported to express NMDARs including both NMDAR1 and NMDAR2A subunits^12^. We used flow cytometry to examine the expression of NMDARs to confirm the presence and maturation of NMDARs in mouse 6–3 microglial cells. We observed that both NMDAR1 and NMDAR2A were expressed in mouse 6–3 microglial cells (n = 4 obtained from 4 independent experiments each; Fig. S1a,b).

We next measured the effect of pretreatment with MK801, another antagonist of NMDARs, on the TNF-induced production of intracellular NO in mouse 6–3 microglial cells. We observed pretreatment of MK801 (10 µM; 12 h) did not affect the amount of TNF-induced increase in the DAF-2 fluorescence at 15 min after a TNF-treatment in mouse 6–3 microglial cells (0.428 ± 0.033, n = 126 cells in control; 0.465 ± 0.028, n = 178 cells in MK801; p = 0.12; Fig. S2a–c). In addition, we also observed that MK801 could not affect the elevation of [Ca^2+^]i induced by TNF in both mouse primary microglial cells (92.8 ± 7.7 nM, n = 137 cells in control; 94.1 ± 7.9 nM, n = 132 cells in MK801; p = 0.41; Fig. S2d,e) and mouse 6–3 microglial cells (not shown). Lastly, we observed that pretreatment with 10 μM MK-801 did not affect the amount of β-Amyloid (1–42) phagocytosed by mouse primary microglial cells (n = 165 cells in control; n = 198 cells in MK801 from 5 independent experiments each; Fig. S2f).

## Discussion

In the present study, we observed that pretreatment with memantine did not affect either production of NO or intracellular Ca^2+^ elevation induced by TNF in rodent microglial cells. Pretreatment with memantine did not affect the mRNA expression of either pro-inflammatory (TNF, IL-1β, IL-6 and CD45) or anti-inflammatory (IL-10, TGF-β and arginase) phenotypes in mouse primary microglial cells. In addition, pretreatment with memantine did not affect the amount of β-Amyloid (1–42) phagocytosed by mouse primary microglial cells. Moreover, we observed that pretreatment with memantine did not affect 11 major proteins, which mainly function in the phagocytosis, degradation of β-Amyloid (1–42) and/or intercellular signaling, including TREM2, DAP12 and neprilysin, in mouse 6–3 microglial cells. To the best of our knowledge, this is the first report to test the direct effects of pretreatment with memantine on rodent microglial functions.

Excessive and long-term activation of NMDARs induces excitotoxicity, ultimately leading to neurodegeneration^21^. Memantine is shown to prevent excess Ca^2+^ entry through NMDARs induced by treatment with Amyloid-β oligomers (AβOs) in cultured neurons prepared from mice^22^. Microglia also expresses NMDARs and applications of a high concentration of NMDA are shown to release pro-inflammatory cytokines and to induce the death of cortical neurons^12^. In animal models of AD, memantine has been reported to protect neuronal death accompanied by suppressing both the proliferation and activation of microglial cells^13,14^. However, these reports did not show memantine directly affects microglial functions in their examinations. In the present study, we suggest that memantine could not directly modulate the microglial functions including intracellular NO and Ca^2+^ mobilization in rodent microglial cells. In addition, we also examined whether pretreatment of memantine directly affects the related proteins, which mainly function in the phagocytosis of rodent microglial cells using flow cytometry. We observed that pretreatment of memantine did not affect the amount of both TREM2 and DAP12, both of which are important for the phagocytic activity of microglia^6,23–25^. We also observed that pretreatment of memantine did not affect the amount of neprilysin, which is a β-Amyloid (1–42)-degrading enzyme important for the clearance of β-Amyloid (1–42) by microglia^26^. Moreover, we observed that pretreatment with memantine did not affect other eight proteins which mainly function in the phagocytosis, degradation of β-Amyloid (1–42) and/or intercellular signaling in rodent microglial cells (Table 1). Specifically, pretreatment with memantine did not affect the amount of any of the following proteins including CX3CR1, CR3(CD11b/c), CD68, Dectin-1/Clec7a, Prostaglandin E synthase (PTGEs), Suppressor of cytokine signaling 3 (Socs3), ADAM10 and ADAM17. However, we observed that pretreatment with memantine significantly increased the amount of expression of A disintegrin and metalloproteinase with thrombospondin motifs 4 (ADAMTS4) and ATP binding cassette subfamily a member 7 (ABCA7) in mouse 6–3 microglial cells (Table 1). ADAMTS4 is one of metalloproteases to degrade chondroitin sulfate proteoglycans leading to destruction of cartilage during arthritis or spinal cord injury. In addition, ADAMTS4 is shown to increase the number of microglia expressing arginase 1, a marker of anti-inflammatory functions in the brain of mouse model of ischemic stroke^27^. Loss-of-function variants of ABCA7 increases the risk of susceptibility to Alzheimer's disease (AD) in Icelanders^28^. ABCA7 is important for microglial phagocytic activity and deletion of ABCA7 exacerbate the load of Aβ plaque in the cerebral cortex of mouse model of AD^29^. Thus, memantine could directly affect some proteins to augment the function of microglial cells leading to protect neuronal death in animal models of AD. Although we observed that pretreatment with memantine did not affect 11 of 13 major proteins, we cannot conclude that memantine has no direct effects on phagocytosis and degradation of β-Amyloid (1–42) mainly mediated by microglial cells. In addition, we need to be aware that mRNA and protein levels could not match in the same experiments.

Mouse primary microglial cells are reported to express NMDARs including both NMDAR1 and NMDAR2A subunits^12^. We also observed that both NMDAR1 and NMDAR2A were expressed in mouse 6–3 microglial cells we used. In addition, we observed pretreatment of 10 µM MK801, another antagonist of NMDARs, did not affect both production of NO and intracellular Ca^2+^ mobilization induced by TNF in rodent microglial cells. We used 10 µM MK801 because MK801 with the same concentration suppressed the NMDA-induced intracellular calcium responses in mouse primary cells^12^. In the present study, we also observed that pretreatment with MK801did not affect phagocytic activity of mouse primary microglial cells. It requires further research to elucidate whether microglial cells express functional NMDARs. Previous reports show that microglial cells do not express functional NMDARs in the rodent brain^30–32^. We also observed that an application of glutamate did not elevate [Ca^2+^]i in any of rat HAPI microglial cells, mouse 6–3 microglial cells and mouse primary microglial cells (unpublished data). To the best of our knowledge, only two reports have clearly shown the presence of functional NMDARs in microglial cells^12,33^. Unfortunately, the two reports examined the effects of MK801, but not the effects of memantine, on microglial functions. In HEK-293 cells, Gilling et al. have previously reported that the antagonistic effects of memantine are strongly voltage-dependent^34^. Thus, it is possible that voltage-dependency could affect the results of our failure to observe the effects of both memantine and MK801 on microglial cellular functions. We have previously reported that pretreatment with donepezil, an acetylcholinesterase inhibitor, directly modulates the microglial functions, including intracellular NO and Ca^2+^ mobilization and phagocytic ability in rodent microglial cells, through the phosphatidylinositol-3 kinase (PI3K) pathway^16^. It is possible that both memantine and MK801 did not affect the PI3K pathway in rodent microglial cells we used. There are no reports of whether NMDARs activate the PI3K pathway in microglia.

Although we noticed one major limitation of our observation is the reliance on the in vitro work of microglia activation, our in vitro work is compatible with the in vivo studies which reports that memantine improves cognitive dysfunction through the indirect effects rather than direct modulatory effects on microglia^13,14^.

## Conclusions

We herein observed that memantine could not directly modulate intracellular NO and Ca^2+^ mobilization and phagocytic activity in rodent microglial cells. These results could be important to understand the effect of memantine for the treatment of AD.

## Methods

### Materials

The drugs used in the present study include memantine, 4,5-diaminofluorescein diacetate (DAF-2DA), Fura2-AM, human recombinant TNFα, adenosine 5′ triphosphate (ATP) (all from Sigma-Aldrich, St. Louis, MO), (+)-MK801 Maleate (R&D Systems, Inc. MN) and β-Amyloid (1–42), 5-FAM-labeled (AnaSpec Inc., CA). Human recombinant TNFα was diluted with the standard external solution to obtain a final concentration. Memantine was diluted with the standard external solution to obtain a final concentration (5 µM). This memantine concentration is sufficient to antagonize the NMDA receptor-mediated currents in cultured hippocampal neurons^35^ or to prevent neurotoxicity in rat cortical neurons^36^. In addition, we used donepezil at the same concentration in our previous report^16^. Drugs that were insoluble in water were first dissolved in dimethylsulfoxide (DMSO; Wako Pure Chemical Industries, Osaka, Japan), then diluted in the standard external solution. The final concentration of DMSO was always less than 0.1%.

### Rodent microglial cells

Primary microglial cells were prepared from the whole brain of 8-week-old male C57BL/6 J mice (CLEA Japan, Inc., Tokyo, Japan) using magnetic-activated cell sorting as we have reported^16,17^. Mouse brain tissues were dissociated enzymatically with a Neural Tissue Dissociation Kit (Miltenyi Biotec, Auburn, CA) according to the manufacturer’s protocol. Briefly, mouse brain tissues were minced with a scalpel, and pre-warmed enzyme mix solution was added to the tissue pieces. After enzymatic dissociation, dissociated tissues were filtered with a 70-µm pore-size cell strainer, and centrifuged. Pellets were re-suspended in MACS buffer (Miltenyi Biotec, Auburn, CA) supplemented with magnetic myelin removal beads (Miltenyi Biotec, Auburn, CA) and incubated for 15 min. Myelin was removed by magnetic separation using LS columns (Miltenyi Biotec, Auburn, CA). To separate primary microglia, cells were magnetically labeled with CD11b MicroBeads (Miltenyi Biotec). CD11b + cells were isolated by LS columns (Miltenyi Biotec), and isolated cells were cultured with DMEM containing 10% FBS, 1% antibiotics, and 1 ng/mL GM-CSF. The purity of isolated microglia was assessed by immunocytochemical staining for the microglial marker, Iba-1, and > 99% of cells stained positively.

The 6–3 microglial cells were established from neonatal C57BL/6J (H-2b) mice as described previously^22,37^. The 6–3 cells were cultured in Eagle’s MEM supplemented with 0.3% NaHCO_3_, 2 mM glutamine, 0.2% glucose, 10 g/mL insulin and 10% FBS. Cells were maintained at 37 °C in a 10% CO2, 90% air atmosphere. GM-CSF was supplemented into the culture medium at a final concentration of 1 ng/mL, to maintain proliferation of the 6–3 cells. Culture media was renewed twice per week.

The rat microglial cell line, highly aggressive proliferating immortalized (HAPI) cells, was kindly donated by Drs. N. P. Morales and F. Hyodo of Kyushu University (Japan). The cells were cultured in DMEM (low glucose; Invitrogen, Waltham, MA), 5% FBS (Hyclone, Logan, UT), 4 mM glutamine (Invitrogen, Waltham, MA), 100,000 U/L Penicillin G, 100 mg/L streptomycin (Mediatech, Tewksbury, MA), and maintained in 5% CO_2_ at 37 °C as previously reported^16,17^.

All experiments were performed in accordance with the guidelines for the care and use of experimental animals by the Japanese Association for Laboratory Animals Science (1987) and were approved by the Saga University Animal Care and Use Committee and carried out according to the Saga University Animal Experimentation Regulations. In addition, all methods were carried out in accordance with the Saga University Animal Experimentation Regulations. This study was carried out in compliance with the ARRIVE guidelines.

### Intracellular NO imaging

The experiments were performed as described previously^16,17^. The microglial cells were loaded with 10 µM DAF-2DA (4,5-diaminofluorescein diacetate; Sigma-Aldrich, St. Louis, MO), a cell-membrane-permeable dye that binds intracellular NO^18^, for 20 min before measurement. For DAF-2 excitation, the cells were illuminated at a 490-nm wavelength using a computerized system. The signal obtained at 490 nm was previously shown to be, among the excitation wavelengths, quantitatively the largest and most representative of change in intracellular NO^38^. The emitted light was collected at 510 nm using a cooled CCD camera. The intracellular DAF-2 fluorescence intensity (F) was recorded for each pixel within a cell boundary. The ratio (F/F0) of fluorescence intensity was estimated from the intensity of fluorescence recorded prior to stimulation (F0).

### Intracellular Ca^2+^ imaging

Intracellular Ca^2+^ imaging using fura-2 AM was performed as reported previously^16,17,37,39^. In brief, the experiments were performed in the external standard solution (in mM: 150 NaCl, 5 KCl, 2 CaCl_2_, 1 MgCl_2_, 10 glucose and 10 HEPES, pH 7.4 with Tris-OH) at room temperature (27 °C). For fura-2 excitation, the cells were illuminated at two alternating wavelengths, 340 and 380 nm, using a computerized system for a rapid dual wavelength Xenon arc. The emitted light was recorded at 510 nm using a cooled CCD camera (Hamamatsu Photonics, Japan). The intracellular Ca^2+^ concentration [Ca^2+^]i was calculated from the ratio (R) of fluorescence recorded at 340 and 380 nm excitation wavelengths for each pixel within a microglial cell boundary. Calibrations (conversion of R340/380 values into calcium concentrations) were performed as described previously^16,17,37,39^, using a Fura-2 calcium imaging calibration kit (Molecular Probes, Eugene, OR). Basal [Ca^2+^]i was determined from the initial 12 images of each cell recording. A [Ca^2+^]i signal was defined as an increase in R 340/380 with clear time correlation to the application of TNF. An increase of [Ca^2+^]i in response to TNF was calculated as the difference between basal [Ca^2+^]i and values obtained at 15 min after treatment with TNF. We tested the effect of 100 µM ATP on rodent microglial cells at the end of all experiments and used cells that showed transient intracellular Ca^2+^ elevation for analysis. All data presented were obtained from at least five dishes and three different cell preparations.

### Quantitative real time-polymerase chain reaction (qRT-PCR)

qRT-PCR was performed using a LightCycler 480 system (Roche Diagnostics, Mannheim, Germany) as previously reported^16^. The mouse primary microglial cells were pre-treated with memantine (5 µM) for 12 h. Cells were washed and the total RNA was extracted using a High Pure RNA Isolation kit (Roche Diagnostics) according to the manufacturer’s protocol, and was subjected to cDNA synthesis using a Transcriptor First Strand cDNA Synthesis kit (Roche Diagnostics). qRT-PCR was performed with primers (TNF: 5′-CTGTAGCCCACGTCGTAGC-3′, 3′-TTGAGATCCATGCCGTTG-5′; CD45: 5′-TCAGAAAATGCAACAGTGACAA-3′, 3′-CCAACTGACATCTTTCAGGTATGA-5′; IL-1β: 5′-AGTTGACGGACCCCAAAAG-3′, 3′-AGCTGGATGCTCTCATCAGG-5′; IL-6: 5′-GCTACCAAACTGGATATAATCAGGA-3′, 3′-CCAGGTAGCTATGGTACTCCAGAA-5′; IL-10: 5′-CAGAGCCACATGCTCCTAGA-3′, 3′-TGTCCAGCTGGTCCTTTGTT-5′; TGF-β: 5′-TGGAGCAACATGTGGAACTC-3′, 3′-GTCAGCAGCCGGTTACCA-5′; Arginase: 5′-GAATCTGCATGGGCAACC-3′, 3′-GAATCCTGGTACATCTGGGAAC-5′). Actin-β of Universal Probe Library (Roche Diagnostics) was used as a house-keeping control gene. All of these primers were used in our previous report^16^. The value of mRNA expression for each sample was automatically calculated as the Ratio by a LightCycler 480 system (Roche Diagnostics, Mannheim, Germany). Ratio = 2^−ΔCp^, ΔCp = Cp(target) − Cp(reference).

### Phagocytosis assay

Phagocytosis was examined via FSX100 Bio Imaging Navigator (Olympus Waltham, MA) using a Human β-Amyloid (1–42), 5-FAM-labeled according to the manufacturer’s protocol. Primary microglial cells cultured in glass-based dishes were used. Human β-Amyloid (1–42), 5-FAM-labeled was reconstituted and diluted with 1X PBS and to a concentration of 3 µg/mL according to both the manufacturer’s protocol and previous reports^40,41^. We incubated the cells in standard culture conditions for 3 h. After aspirating the culture medium, the cells were fixed with 4% paraformaldehyde. Then, after discarding paraformaldehyde, we washed fixed cells with 1 mL phosphate buffered salts (PBS) twice and measured the fluorescence intensity of FAM along the long axis of the cytoplasm using an Imaging Navigator.

### Flow cytometry

Flow cytometry was performed using a FACSVerse Flow Cytometer (BD Biosciences, San Jose, CA), as previously reported^16^. Flow cytometry data were analyzed using FlowJo v10.6.1 (BD Life Sciences Informatics, Ashland, OR). The mouse 6–3 microglial cells were harvested by nonenzymatic cell dissociation solution (Sigma) and a cell lifter (Corning). The cells were fixed with 4% paraformaldehyde and permeabilized with 0.1% Triton X-100. After blocking of Fc receptors by FcR Blocking Reagent, mouse (Miltenyi Biotec, Auburn, CA), the cells were stained with each antibody and the fluorescence intensity of the cells was measured from 9 to 12 dishes (10,000 cells/dish) in each antibody condition. All antibodies used for flow cytometry are listed in Table 2.

**Table 2:** Antibodies used for flow cytometry.

Antigen protein	Antibody
ADAMTS4	Anti-ADAMTS4 polyclonal antibody, ALEXA FLUOR 555 conjugated bs-4191R-A555 BIS
ABCA7	Anti-ABCA7 polyclonal antibody, ALEXA FLUOR 647 conjugated bs-11180R-A647 BIS
TREM2	Anti-TREM2 polyclonal antibody, ALEXA FLUOR 647 conjugated bs-2723R-A647 BIS
DAP12	Anti-DAP12 polyclonal antibody, FITC conjugated bs-12630R-FITC BIS
Neprilysin	Anti-neprilysin/CD10 polyclonal antibody, ALEXA FLUOR 647 conjugated bs-0527R-A647 BIS
CX3CR1	Anti-CX3CR1 polyclonal antibody, FITC conjugated bs-1728R-FITC BIS
CR3(CD11b/c)	AntiCD11b/c polyclonal antibody, PE conjugated bs-1014R-PE BIS
CD68	Anti-CD68 polyclonal antibody, FITC conjugate bs-1432R-FITC BIS
Clec7a/Dectin-1	Anti-Beta glucan receptor (Clec7a/Dectin-1) polyclonal antibody, ALEXA FLUOR 555 conjugated bs-2455R-A555 BIS
PTGEs (Prostaglandin E synthase)	Anti-PTGEs (prostaglandin E synthase) polyclonal antibody, FITC Conjugated, bs-1880R-FITC BIS
Socs3 (suppressor of cytokine signaling 3)	Anti-Socs3 polyclonal antibody, FITC conjugated A61096-100 EPG
ADAM10	Anti-ADAM10 polyclonal antibody, ALEXA FLUOR 647 conjugated bs-3574R-A647 BIS
ADAM17	Anti-ADAM17 polyclonal antibody, PE conjugated bs-4236R-PE BIS
NMDAR1	Anti-NMDAR1 (Ser897) polyclonal antibody, FITC conjugated bs-3903R-FITC BIS
NMDAR2	Anti-NMDAR2A polyclonal antibody, ALEXA FLUOR 647 conjugated bs-3507R-A647 BIS

### Statistics

All statistical analyses were performed with Statistical Package for the Social Sciences (SPSS) software (version 18.0; SPSS Inc., Chicago, IL). All quantified data represent a mean ± SEM. Statistical significance was determined by ANOVA and Tukey’s post hoc test when more than two groups were compared, and Student’s t test when one group was compared with the control group. p < 0.05 was considered significant.

## Supplementary Information

Supplementary Information.
