# Supplementary material for: Effect of memantine, an anti-Alzheimer’s drug, on rodent microglial cells in vitro
Source: Sci Rep. 2021 Mar 17;11:6151. doi: 10.1038/s41598-021-85625-4 (PMC7969939; doi:10.1038/s41598-021-85625-4)
Supplement: Supplementary file 1 — Supplementary Information. [file 41598_2021_85625_MOESM1_ESM.pdf]

Supplementary information

Title:

Effect of memantine, an anti-Alzheimer's drug, on rodent microglial cells in vitro

Authors:

Toru Murakawa-Hirachi[1]*, Yoshito Mizoguchi[1]*, Masahiro Ohgidani[2], Yoshinori Haraguchi[1] and Akira Monji[1]

1. Department of Psychiatry, Faculty of Medicine, Saga University, 5-1-1 Nabeshima, Saga 849-8501, Japan

2. Department of Integrative Anatomy, Nagoya City University Graduate School of Medical Sciences, Nagoya 467-8601, Japan

*T. Murakawa and Y. Mizoguchi have contributed equally to this work.

Corresponding authors:

Yoshito Mizoguchi, M.D., Ph.D.

Department of Psychiatry, Faculty of Medicine, Saga University, 5-1-1 Nabeshima, Saga 849-8501, Japan.

Phone: +81-952-34-2304 Fax: +81-952-34-2048

E-mail: ymizo@cc.saga-u.ac.jp

Supplemental Figure 1.

Expression of NMDARs in rodent microglial cells. We observed that both NMDAR1 (A) and NMDAR2A (B) were expressed in mouse 6-3 microglial cells using flow cytometry. In each panel, ‘Non-stain’ and ‘Control’ show measurements without antibody and with antibody alone, respectively.

Supplemental Figure 2.

Effects of pretreatment with MK801, another antagonist of NMDARs, on production of NO, intracellular Ca^{2+} elevation and phagocytic activity in rodent microglial cells. **A.B.** Ten representative traces showing the treatment of 0.1 ng/mL TNF induced the increase in the DAF-2 fluorescence (A) and 12 hrs pretreatment with 10 μM MK801 did not affect the TNF-induced increase in the DAF-2 fluorescence in mouse 6-3 microglial cells (**B**). **C.** Bar graphs showing that pretreatment with MK801 (10μM, 12 hrs) did not the production of NO induced by TNF treatment in mouse 6-3 microglial cells. **D.** Average traces of 5 $[Ca^{2+}]$i traces showing a treatment of 3 ng/mL TNF-induced sustained increase in $[Ca^{2+}]$i in mouse primary microglial cells. **E.** Bar graphs showing that pretreatment with MK801 (10μM, 12 hrs) did not affect the elevation of $[Ca^{2+}]$i induced by TNF in mouse primary microglial cells. **F**. Pretreatment with MK801 (10μM, 12 hrs) did not affect phagocytic activity of mouse primary microglial cells. Bar graph showing pretreatment of 10 μM MK801 for 12 hrs did not affect the amount of β-Amyloid (1-42) phagocytosed by mouse primary microglial cells (n= 165 cells in control; n= 198 cells in MK801 from 5 independent experiments each). NS: not significant vs control.

Figure S1

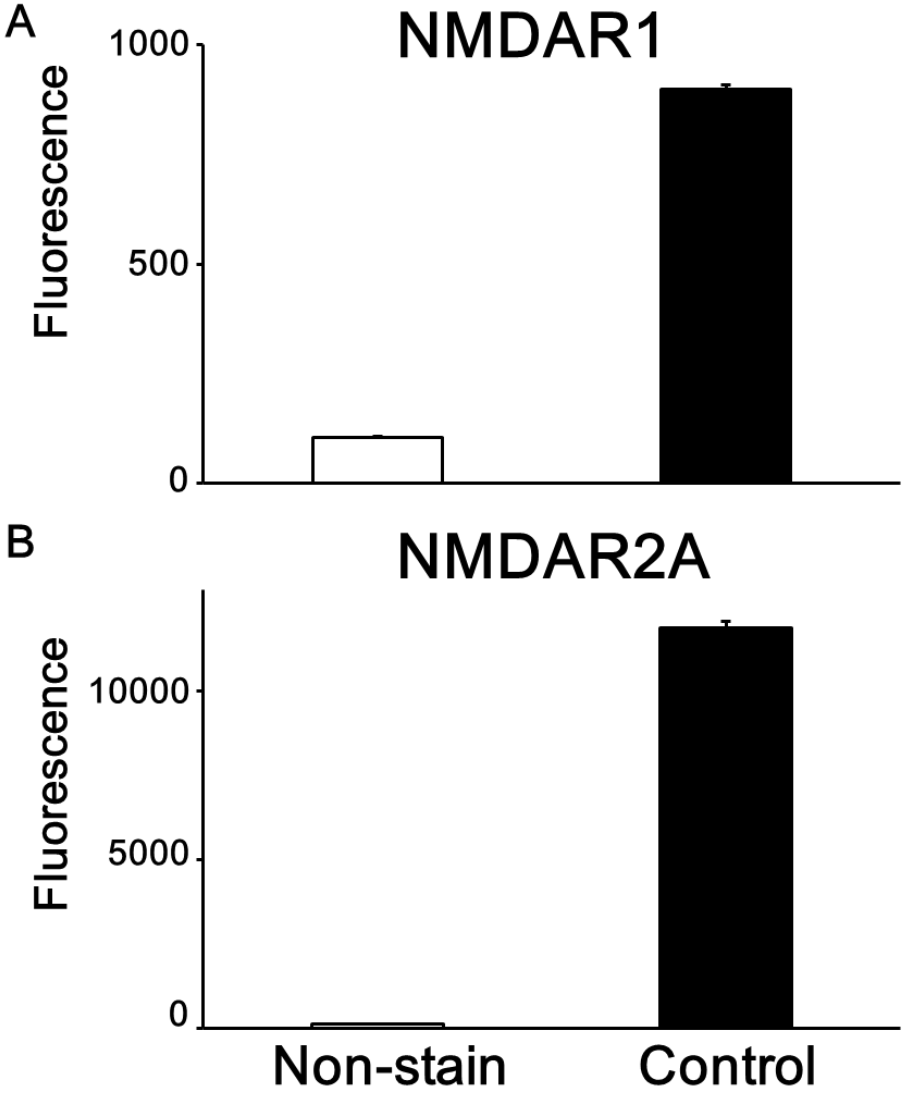
A
NMDAR1
1000
500
0
Fluorescence
B
NMDAR2A
10000
5000
0
Fluorescence
Non-stain
Control

Figure S2

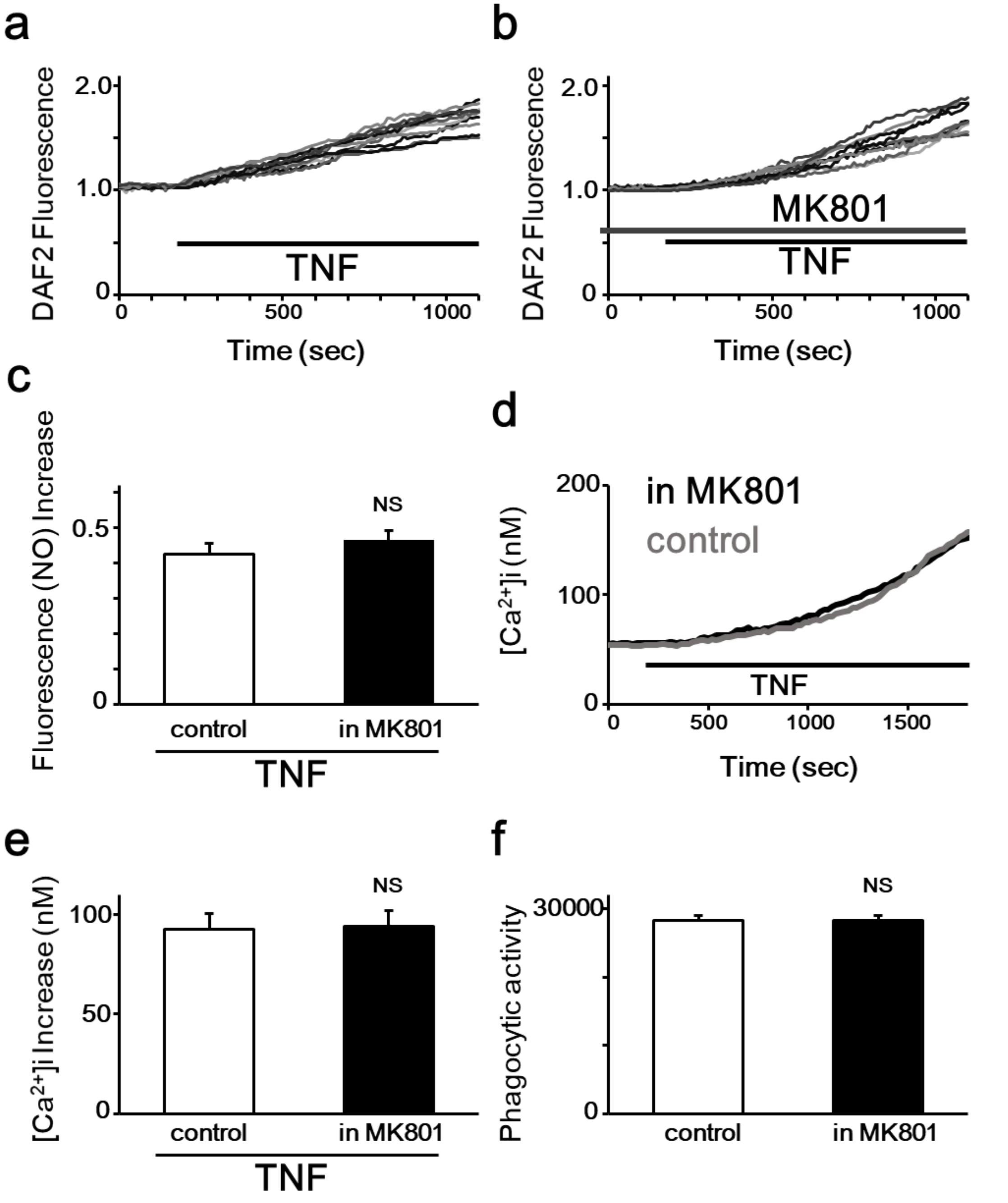
a
DAF2 Fluorescence
2.0
1.0
0
TNF
0
500
1000
Time (sec)
b
DAF2 Fluorescence
2.0
1.0
0
MK801
TNF
0
500
1000
Time (sec)
c
Fluorescence (NO) Increase
0.5
0
NS
control
in MK801
TNF
d
[Ca2+]i (nM)
200
100
0
in MK801
control
TNF
0
500
1000
1500
Time (sec)
e
[Ca2+]i Increase (nM)
100
50
0
NS
control
in MK801
TNF
f
Phagocytic activity
30000
0
NS
control
in MK801
